# Fiber–Sample Distance, An Important Parameter To Be Considered in Headspace Solid-Phase Microextraction Applications

**DOI:** 10.1021/acs.analchem.9b05386

**Published:** 2020-05-08

**Authors:** Franks Kamgang Nzekoue, Simone Angeloni, Giovanni Caprioli, Manuela Cortese, Filippo Maggi, Umberto Marini Bettolo Marconi, Andrea Perali, Massimo Ricciutelli, Gianni Sagratini, Sauro Vittori

**Affiliations:** ^†^School of Pharmacy and ^‡^HPLC-MS Lab, University of Camerino, via Sant’Agostino 1, 62032 Camerino, Italy; § School of Sciences and Technology, University of Camerino, Via Madonna delle Carceri, 62032 Camerino, Italy

## Abstract

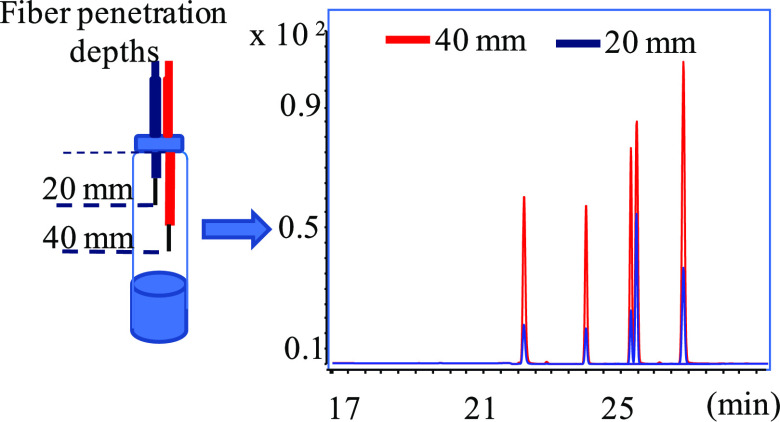

To define and control the parameters which impact headspace solid-phase microextraction (HS-SPME), it is important to reach the highest level of reproducibility. The present study aims to assess, for the first time, the effect of fiber–sample distance during HS-SPME in pre-equilibrium conditions. Analyses were primarily performed on mixtures of standard volatiles compounds (alkanes, alcohols, organic acids) designed in our lab and then on various food matrices (wine, chicken, cheese, tea), repeating already published experiments. Extractions were performed varying fiber penetration depths (10–60 mm) at different times (10–60 min) and temperatures of extraction (30–80 °C). The study revealed that variation of the distance between the fiber and the sample into the vial clearly impacts the results obtained during HS-SPME when conditions are such that no equilibrium is reached in HS. For example, in wine analysis, the percentage of octanoic acid at 80 °C was higher at 40 mm (7.5 ± 0.2%) than that at 20 mm (4.4 ± 0.3%). Moreover, regardless of the extraction temperature, the lower the time of extraction, the stronger the dependence on the fiber–sample distance. Indeed, at 60 °C, the obtained response factors for octadecane at 20 and 40 mm of fiber penetration were 21.8 and 44.5, respectively, after 10 min of extraction, 54.1 and 71.0 after 30 min, and 79.4 and 82.4 after 60 min of extraction. The analyses have been here corroborated by a theoretical model based on the diffusion equation. Therefore, to improve the method robustness during HS-SPME studies, we suggest specifying the fiber penetration depth or the fiber–sample distance with the other parameters of extraction.

Headspace solid-phase microextraction (HS-SPME) is one of the major extraction techniques in volatile organic compound (VOC) analysis.^1^ This technique, introduced in 1990 by Janusz Pawliszyn,^1^ is nowadays commonly applied on food,^2^ environmental,^3^ and biological samples^4^ due to its operation simplicity, rapidity, environmentally friendly impact, and reusability of tools and equipment.^5^ HS-SPME is based on the transfer of VOCs from the sample matrix to the headspace of a closed container in which the sample is introduced, followed by their absorption by a SPME fiber coating.^6^ The level of absorption is influenced by many parameters, such as the initial concentrations of the analytes, their constant of distribution, the fiber coating volume, the sample volume, the temperature, and the time of extraction.^7,8^

Time and temperature of extraction are determinant in HS-SPME, since in a sample all analytes do not have the same distribution constant, and therefore, the VOCs composition in the headspace (HS) can change with time. After a certain time, the distribution of analytes from the sample to the fiber coating can reach an equilibrium state where the concentrations of volatiles compounds are homogeneous in the sample matrix; the HS and the coating phases and do not change further.^6^ Higher temperatures are generally used to speed up the mass transfer and reduce the time to reach equilibrium. This time is very important since it allows one to have a homogeneous mass transfer and analytes composition in all of the volume of the HS. In other words, during SPME, molecules with lower distribution ratios (*K*) and higher diffusion constants (*D*) reach equilibrium faster.^9,10^ This may mean that in pre-equilibrium conditions the VOCs composition is not the same over the entire depth of the HS.

In most HS-SPME applications, the equilibrium may be not reached or even assessed for different reasons. This can be seen, for example, when time is shortened as much as possible to have faster extraction and analytical methods and, therefore, increase sample throughput^11^ or when extractions are performed at low temperatures to reproduce natural conditions or protect thermolabile compounds. In all of these cases, extractions could happen in pre-equilibrium conditions; this is not a problem as long as extraction conditions are kept the same during each sampling.^12^

Among the parameters to be maintained unchanged in pre-equilibrium conditions, much attention is given to the extraction time, the speed of agitation, and the temperature. However, to our knowledge, no attention has been given until now to the SPME fiber penetration depth into the vial as a parameter which can affect SPME results. Indeed, in published papers dealing with HS-SPME methods, the authors just mention that the SPME fiber was exposed to the sample HS without specifying the fiber penetration depth or the distance between the surface of the sample and the fiber.^13^ This can suppose that either it is proven that they are working in equilibrium conditions or there is no effect of the fiber penetration depth on HS-SPME in pre-equilibrium conditions. This is precisely what we wanted to know because of the lack of homogeneity of the HS in pre-equilibrium conditions.

In simple words, we wanted to assess if before the equilibrium the extraction results could be similar whether the SPME fiber is exposed near or far from the sample matrix surface. Indeed, the robustness of an analytical method is a determinant parameter to be ensured during method development and validation.^14^ Thus, it could be very important to assess if the fiber penetration distance may impact the HS-SPME because, in general practice, the position of the fiber is random and, consequently, the obtained results could be different from lab to lab or operator to operator.

Therefore, the aim of the present study was to assess the effect of the fiber–sample distance on the quantitative and qualitative results of HS-SPME. This was done by carrying out a number of different analyses, comparing the results obtained at different fiber penetration depths on (a) mixtures of standard compounds at different times and temperatures of extraction and (b) food sample matrixes, reproducing various HS-SPME methods reported in the literature. Moreover, this study aims also at setting up a new formula, which will define more in detail the dynamic of volatile compounds during HS-SPME.

The results of this study could be crucial in order to know whether the fiber penetration depth or the fiber–sample distance is a parameter to be considered in HS-SPME analyses.

## Experimental Section

### Concepts of Fiber–Sample Distance and Fiber Penetration Depth

To perform HS-SPME experiments, samples were introduced in 20 mL (23 × 75 mm) HS vials sealed with an 18 mm HS screw cap (Phenomenex, Torrance, CA, USA) and stirred at 250 rpm; vial volume and stirring speed were defined according to the most common conditions reported in 200 HS-SPME papers published in 2019 (Table S1). The fiber–sample distance represents the distance between the sample surface and the top of the SPME fiber, while the fiber penetration depth is measured as the distance between the screw cap and the tip of the exposed SPME fiber. Fiber–sample distance depends on the volume of sample placed in the HS vial and the fiber penetration depth. In order to simplify the understanding of the study, the fiber penetration depth was reported in some sections of the article instead of the fiber–sample distance.

### HS-SPME Experimental Conditions

In order to study the effect of the fiber–sample distance, different depths of fiber penetration were compared: 10, 20, 30, 40, and 50 mm. Stirring rate and vial volume were kept for all experiments at 250 rpm and 20 mL, respectively, as stated above. Analyses were performed on mixtures of standard samples and on complex food matrixes comparing, in various conditions, different fiber penetration depths.

### Analyses of Mixtures of Standard Compounds

#### Studies on Alkane Standards

The first study examined the quantitative results obtained during HS-SPME on 2 alkanes, pentane and octadecane, varying the fiber penetration depth into the vial. To perform this study, various extractions were carried out at different times (10, 30, and 60 min) and temperatures (40, 60, and 80°C). Briefly, 1 mL of an aqueous standard solution containing the two alkanes at a concentration of 100 μg mL^–1^ was placed in a HS vial. The vial was then sealed with a screw cap and introduced in the HS heater. After 10 min of incubation, a 50/30 μm divinylbenzene/carboxene/polydimethylsiloxane (DVB/CAR/PDMS) SPME fiber was exposed to the sample HS at penetration depths of 20 and 40 mm. The results obtained from the different penetration depths were compared at different times and temperatures of extraction.

#### Studies on a Standard Mixture of Six VOCs

The second study consisted in comparing the quantitative results of HS-SPME on mixtures of 6 VOCs: 3-methylbutanal, hexen-1-ol, furfural, furfuryl acetate, linalool, and guaiacol. Analyzed samples consisted of mixtures of standard compounds (1 mL) at a concentration of 100 μg mL^–1^ in water. Different extractions were performed in the conditions reported above, varying the extraction temperatures (40, 60, and 80 °C) and maintaining the same time of extraction (15 min). An 85 μm polyacrylate (PA) fiber (Supelco, Bellefonte, PA, USA) was used for HS extraction. The peak areas of the different analytes were compared at the 2 penetration depths studied (20 and 40 mm).

#### Studies on Free Fatty Acids Mixtures

Analyses were also carried out on mixtures of 4 free fatty acids (FFAs): butanoic, hexanoic, octanoic, and decanoic acids. Extractions were performed according to the method developed by Nzekoue et al.^15^ Briefly, 2 mL of a 10 μg mL^–1^ mixture standard solution was placed in the HS vials with 0.2 g of NaH_2_PO_4_ and incubated at 60°C. Isovaleric acid was used as internal standard (10 μg mL^–1^). After 30 min of incubation, a 75 μm carboxene/polydimethylsiloxane (CAR/PDMS) (Supelco, Bellefonte, PA, USA) was exposed at 2 exposition distances (20 and 40 mm) for 20 min.

### Analyses on Food Samples

Different HS-SPME methods reported in the literature were applied in order to assess the effect of the fiber–sample distance by changing the penetration depth of the fiber.

#### Studies on Wine

HS-SPME were performed on 3 mL of an Italian white wine named “*primo fiore*” (Camerino, Italy) introduced in the HS vial.^16^ Samples were incubated at different temperatures (30, 50, and 80 °C) and at a fixed time of fiber exposition (20 min). Used SPME fiber was a 50/30 μm DVB/CAR/PDMS. Fiber was exposed at 2 distances of penetration (20 and 40 mm). The obtained results were expressed in terms of peak area percentages (%) of the identified VOCs and were compared.

#### Studies on Cheese

Extractions were carried out following the same method conditions reported by Guarrasi et al.,^17^ varying the fiber exposition distance. Briefly, 2 g of caciocavallo cheese (Camigliatello Silano, Italy) was placed in a HS vial and incubated at 45 °C for 5 min. Then a 75 μm CAR/PDMS fiber was exposed to the HS for 30 min at 2 fiber penetration depths (20 and 40 mm).

#### Studies on Tea

Following the method reported by Lin et al.,^18^ HS-SPME was performed at 2 fiber penetration depths (20 and 40 mm). Briefly, 1 g of tea was inserted into a vial, and then a PDMS/DVB fiber was directly exposed to the HS at 50 °C for 40 min.

#### Studies on Chicken

The conditions of HS-SPME were those reported by Argyri et al.,^19^ varying the fiber penetration depths (20 and 40 mm). Briefly, 2 g of ground chicken meat was placed in a HS vial and incubated at 40 °C in a heat agitator for 15 min. Then a 50/30 μm DVB/CAR/PDMS was exposed to the HS for 30 min.

### Theoretical Method

To investigate the effect of the fiber–sample distance in the pre-equilibrium regime, we studied the changes of the concentration of the diffusing analytes as a function of the fiber penetration depth and the extraction time. Such a process is governed by Fick’s law, namely, a partial differential equation describing the unsteady diffusion of the analyte in the HS. The theoretical method employed is that illustrated by Truskey et al.^20^

### Statistical Analysis

All of the analyses were performed in triplicate (*n* = 3 biological replicates), and the results are reported as the mean ± standard deviation (mean ± S.D). Details are reported in the Supporting Information.

## Results and Discussion

### Analyses of Standard Compounds Mixtures

To study the effect of the fiber–sample distance, trials were first performed on a mixture of standard compounds. In fact, working with standards was ideal to assess with accuracy how the variation of fiber penetration could impact the results obtained during HS-SPME. Therefore, different mixtures of VOCs were studied in various conditions.

#### Studies on a Pair of Alkane Standards

**Figure 1:**
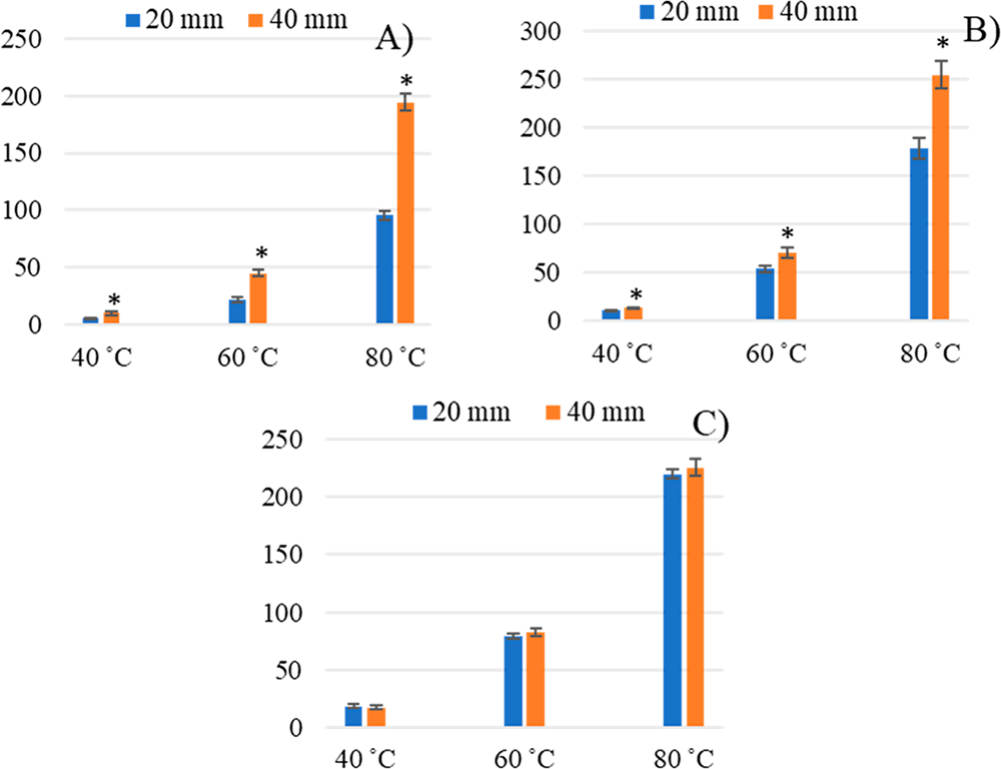
Analyses of the pair of alkanes: response factors (RF) obtained at two fiber penetration depths (20 and 40 mm) at various conditions of extraction. (A) Fiber exposition time: 10 min. (B) Fiber exposition time: 30 min. (C) Fiber exposition time: 60 min. (RF= 10 × octadecane peak area/pentane peak area). (*) Data were significant for *p* <  0.05.

The first study was performed on a simplified model made of two VOCs of the same chemical class with molecular weight (MW) difference. In this context, pentane and octadecane were chosen and mixtures of these two compounds were analyzed at different temperatures and times of extraction. Figure 1 shows the response factors (RF= 10 × octadecane peak area/pentane peak area) obtained comparing two fiber penetration depths (20 and 40 mm) at different conditions of extraction. Whatever the SPME temperature, we can note that the lower the time of extraction, the higher the RF differences between 20 and 40 mm. Indeed, after 10 and 30 min of extraction, the RF was higher at 40 mm than at 20 mm (*p* < 0.05). However, after 60 min, there was no statistically significant difference. These results could be explained by the volatility differences between the two molecules. Indeed, being less volatile and thus having a lower rate of diffusion, octadecane may reach equilibrium more slowly than pentane. This implies that in pre-equilibrium conditions the concentration of octadecane is lower in the upper part of the HS, while it is higher in the lower part, which is closer to the sample surface. Therefore, under nonequilibrium conditions, the extracted amount of octadecane is higher in the bottom regions of the HS, which is sampled in correspondence of deeper fiber penetration (40 mm), and consequently, the RF appears to be increased.

Furthermore, this explanation is supported by the fact that the RF increased also with temperature increment. Indeed, higher temperatures are often used in HS-SPME to accelerate the mass transfer of VOCs, and the increase of RF reflects an increase of octadecane transfer in HS and confirms its lower volatility with respect to pentane. Moreover, the nonstatistically significant differences observed after 60 min reveals that the equilibrium was reached, and thus, the analyte composition was similar in all of the HS volume.

After observing differences in this simplified model, comparisons of fiber penetration depths were performed in more complex models.

#### Studies on a Mixture of Six VOCs

**Figure 2:**
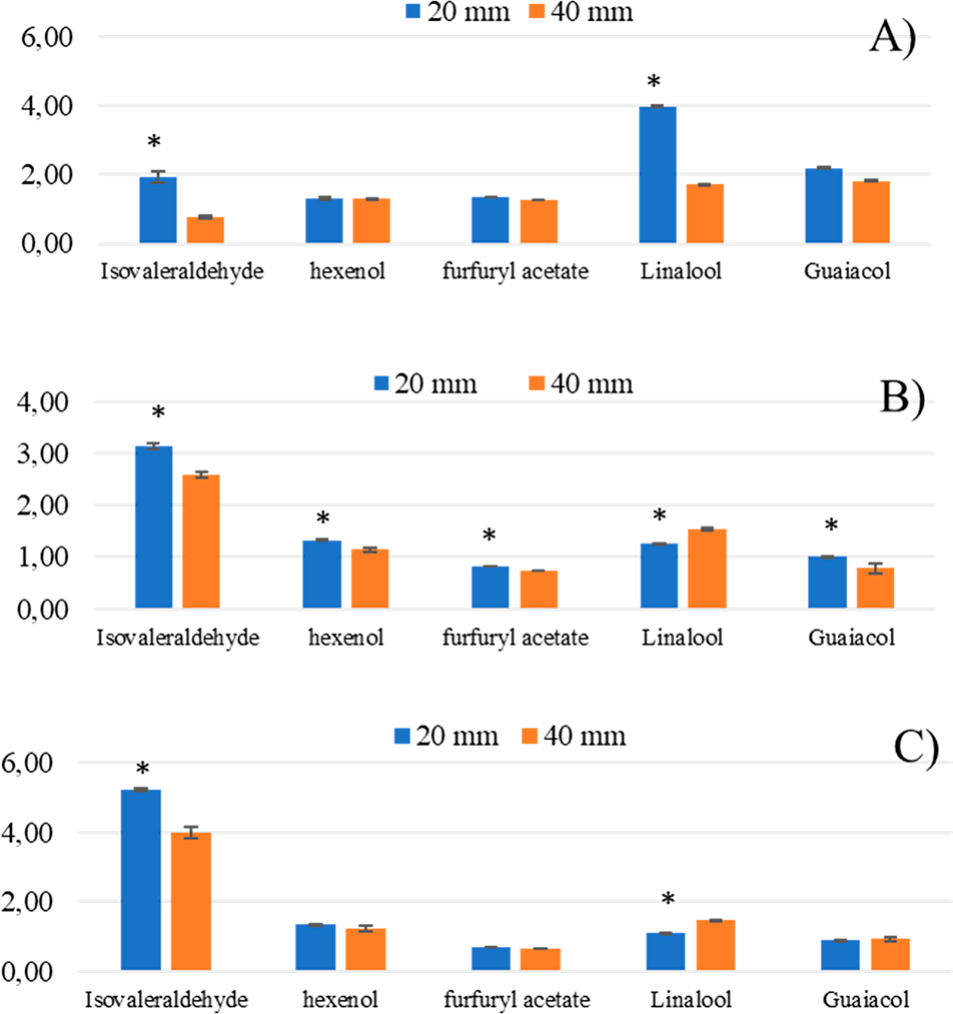
Analyses of a mixture of 6 volatiles organic compounds (VOCs). Response factors (RF) obtained at two fiber penetration distances (20 and 40 mm) at various temperatures of extraction (fixed time of extraction = 15 min). (A) Temperature of extraction: 40 °C. (B) Temperature of extraction: 60 °C. (C) Temperature of extraction: 80 °C. RF = analyte peak area/furfural peak area. (*) Data were significant for *p*  <  0.05.

The first complex model studied was made of 6 organic compounds of different chemical classes such as alcohols (hexen-1-ol, linalool, guaiacol), esters (furfuryl acetate), and aldehydes (isovaleraldehyde, furfural) with a wide MW range (85.13–154.25 g mol^–1^). Figure 2 shows the comparison of 2 depths of fiber penetration (20 and 40 mm) at different temperatures (40, 60, and 80 °C), and the results are expressed as RF using furfural as an internal standard (RF = analyte peak area/furfural peak area). The sample stirring rate was maintained at 250 rpm.

Obtained results showed a gap between the RF of analytes at 20 and 40 mm of penetration. These gaps were statistically significant (*p* < 0.05) for isovaleraldehyde and linalool. For these 2 compounds, we noted a reduction of the gaps with the temperature increment, though the differences remained statistically significant (*p* < 0.05).

Moreover, comparing the peak areas of analytes, we note that the quantity of analytes absorbed by the fiber at 40 mm depth was higher than that at 20 mm (Figure 4A). Except for isovaleraldehyde at 80 °C, these differences of absorbed amounts were statistically significant for all analytes at all temperatures (*p* ≤ 0.05) (Figure S1).

Furthermore, comparisons were performed at higher stirring rates: 400 and 550 rpm. At 400 rpm, the difference remained significant for 3 of the 5 VOCs, while at 550 rpm, the difference remained significant for isovaleraldehyde (Figure S2).

Therefore, although the increment of temperature and stirring rate can speed up the mass transfer, it was not enough during short extraction times to overcome the heterogeneity of the HS. These results contribute to confirm the impact of fiber–sample distance on HS-SPME experiments performed in pre-equilibrium conditions.

#### Studies on Free Fatty Acids Mixtures

**Figure 3:**
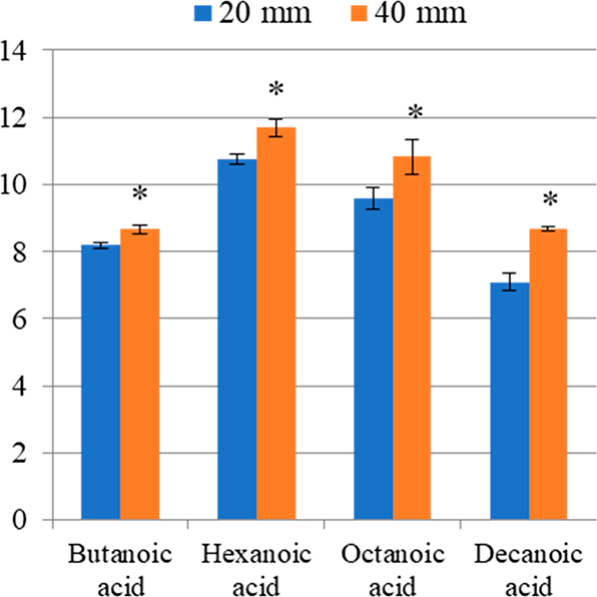
Analyses of a mixture of 6 volatiles organic compounds (VOCs). Peak area of each VOC obtained at two fiber exposition depths (20 and 40 mm) at various temperatures of extraction (40, 60, and 80 °C). (*) Data were significant for *p* <  0.05.

Another complex model was made of 4 free fatty acids and studied to compare the HS-SPME results at 40 and 20 mm penetration. Isovaleric acid was used as internal standard, and RF (RF = analyte peak area/isovaleric acid peak area) was calculated for each analyte. After 30 min of incubation, followed by 20 min of extraction at 60 °C, it was observed that the RF at 40 mm was higher (*p* ≤ 0.05) than that at 20 mm for each of the 4 analytes considered (Figure 3). These results highlight once again the importance to report the fiber penetration depth or the fiber–sample distance during HS-SPME analyses in order to improve the method robustness.

After observing these statistically significant differences on standard samples, it was therefore necessary to perform similar experiments on real sample matrices in order to evaluate further illustrations of the impact of sample–fiber distance on HS-SPME results.

### Studies on Food Samples

HS-SPME is commonly applied in food analysis to study VOCs profile or to quantify specific compounds.^21,22^ It is important to note that, generally, in articles in which HS-SPME is used, no information is given on the distance between the fiber and the sample surface. Besides, many studies are performed with short extraction time;^23^ therefore, those could be under pre-equilibrium conditions. In this perspective, different food matrices were studied, reproducing extraction methods reported in the literature but at different fiber penetration depths.

#### Wine

Samples of “*primo fiore*” wine were analyzed after 20 min of HS-SPME at different temperatures (30, 50, and 80 °C). Table S3 shows a comparison between the results obtained from two fiber penetration depths (20 and 40 mm). Twenty VOCs were identified, and the results are expressed as peak area percentage for each VOC (% = 100 × peak area of analyte/total peak area).

**Figure 4:**
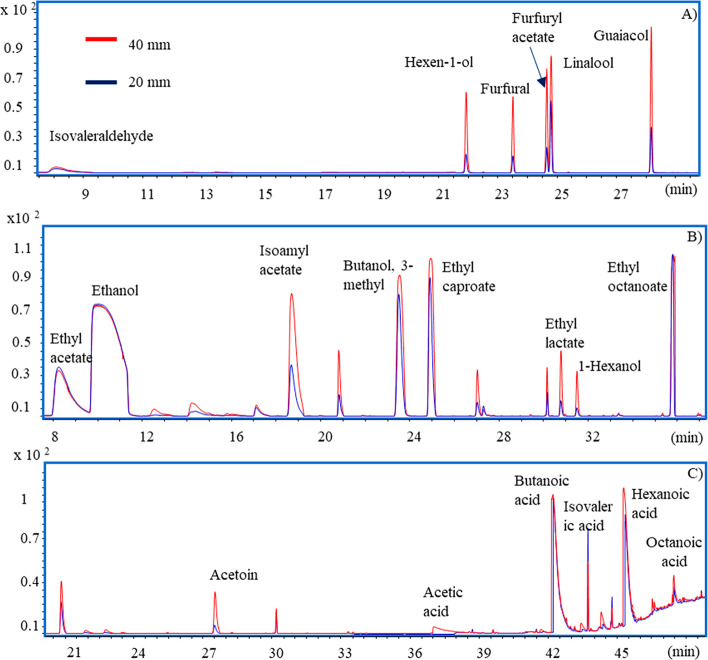
Overlaid chromatograms obtained at 20 and 40 mm of fiber penetration distances. (A) Mix of 6 volatile organic compounds (extraction time = 15 min; extraction temperature = 60 °C). (B) Wine analysis (extraction time = 15 min; extraction temperature = 30 °C). (C) Cheese analysis (extraction time = 30 min; extraction temperature = 45 °C).

At 30 °C, some differences can be observed between the volatile profiles obtained at 20 and 40 mm of penetration. Although ethanol remained the most abundant VOC, its percentage decreased (*p* ≤ 0.05) by bringing the fiber closer to the sample (50.3% vs 39.7%). This decrease was associated with the percentage increment of 15 VOCs such as isoamyl acetate, hexanol, and decanoic acid (Table S3). The same HS-SPME method was repeated on another type of wyne (Tavernello) and similar differences were observed between extraction at 20 and 40 mm (Table S4). This can be explained by the volatility differences of the VOCs. In fact, compounds with low volatility need more time to reach the equilibrium and thus are more concentrated in the lower part of the HS during pre-equilibrium conditions. On the contrary, highly volatile compounds, such as ethyl acetate and ethanol, reach equilibrium faster and thus have the same concentration in all of the HS volume. For example, the absorbed level of ethanol was similar at the 2 distances of fiber exposition, while the level of isoamyl acetate tended to increase at 40 mm (Figure 4B). This proves that the equilibrium was reached for ethanol, while isoamyl acetate, as many other VOCs, was in pre-equilibrium.

The same differences were observed at 50 and 80 °C despite the temperature increment. At 40 mm, we noted a reduction of ethyl acetate and ethanol percentages, while the percentages of other VOCs such as free fatty acids (hexanoic, octanoic, and decanoic acids) increased (Table S3).

In food matrix, each VOC has its diffusion rate and reaches the equilibrium after a specific time. Therefore, in pre-equilibrium conditions, the respective proportion of each compound can vary in the HS. Consequently, it is important to consider fiber penetration during HS-SPME.

#### Cheese

The effect of the fiber–sample distance was assessed on caciocavallo cheese^17^ by testing two fiber penetration depths (20 and 40 mm). Table S5 shows the relative abundance percentages of the identified VOCs at 20 and 40 mm of fiber penetration. It can be clearly seen that the depth variation of fiber exposition had a significant effect not only on the abundance of the VOCs absorbed but also on their relative proportions (Figure 4C). Indeed, from 20 to 40 mm of fiber penetration, we noted a significant (*p* ≤ 0.05) reduction of butanoic acid percentage (59.93% vs 46.72%) and a significant increment (*p* ≤ 0.05) of 9 of the 12 identified compounds. The highest gap of increment was observed with acetic acid, which had a relative proportion of 0.08% at 20 mm, while it reached 4.69% at 40 mm. Pronounced increases were also observed with acetoin (2.16% vs 5.60%), isovaleric acid (0.54% vs 1.27%), and octanoic acid (1.93% vs 3.44%). On the contrary, 3-methyl-1-butanol (0.15% vs 0.19%) and hexanoic acid (32.91% vs 33.32%) levels remained statistically similar. These results proved that the dynamic complexity of VOCs requires one to consider the distance between the fiber and the sample during HS-SPME in pre-equilibrium conditions. Otherwise, the reliability and reproducibility of HS-SPME methods and results should be at stake.

#### Tea

Twelve VOCs were identified after HS-SPME of tea at both 20 and 40 mm of fiber penetration.^18^ The relative percentage of hexanal was lower at 20 mm (24.2 ± 2.3%) than at 40 mm (35.5 ± 1.6%). Statistically significant differences were not observed with other compounds. Moreover, the total peak area of the identified compounds, which is proportional to the levels of VOCs absorbed, tended to be higher at 40 mm (22.7 ± 0.9 × 10^6^) than at 20 mm (19.5 ± 0.4 × 10^6^) of fiber penetration (Table S6).

#### Chicken

Following the HS-SPME conditions reported by Argyri et al.,^19^ 10 VOCs were identified and relative percentages obtained at 20 and 40 mm were compared. As shown in Table S7, statistically significant differences (*p* ≤ 0.05) were observed for 4 of the 10 volatiles (nonane, acetoin, leucic acid, and phenol). Moreover, the total peak areas of the identified compounds were higher (*p* ≤ 0.05) at 40 mm than at 20 mm. These differences confirmed the importance of the fiber–sample distance on HS-SPME analytical results.

### Theoretical Analyses

We wanted to show how the analyte concentration depends on the fiber–sample distance if the system is not at thermodynamic equilibrium. Under these conditions, there is a flux of molecules leaving the bottom region occupied by the liquid solution and diffusing into the headspace. For the sake of simplicity, we assume that the spatial variation of the concentration (*C*) may occur only along the vertical *x* direction and obeys Fick’s equation
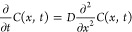
1where *D* is the diffusion coefficient of the analyte in the headspace region. It is assumed that its concentration remains constant in time at the liquid surface located at *x* = 0 (i.e., *C*(0,*t*) = CL) and is initially zero for *x* ≥ 0.

**Figure 5:**
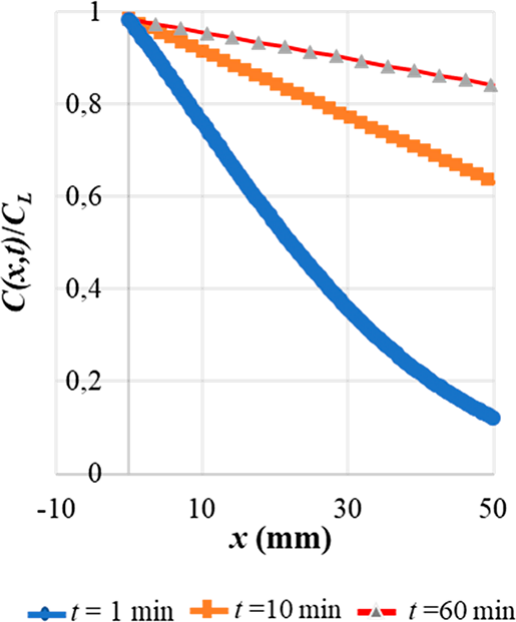
Plot of the concentration *C(x,t)*/*C*_L_ as a function of the fiber–sample distance for extraction times *t* (1, 10, and 60 min). We used the diffusion coefficient of furfural (*D* = 0.0872 cm^2^/s). *C*_L_ = Concentration at the liquid surface; *x* = fiber–sample distance; *t* = time of extraction; *C(x,t)* = concentration as a function of the fiber–sample distance for the extraction time.

**Figure 6:**
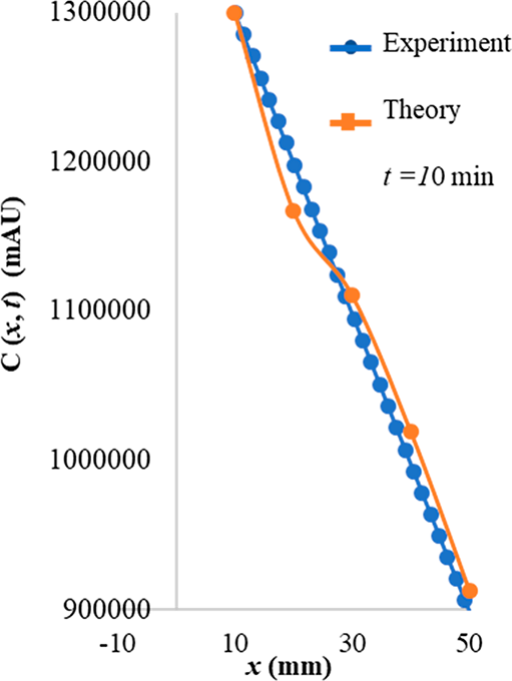
Comparison between the theoretical concentration as a function of the fiber–sample distance for extraction times *t* = 10 min and the experimental HS-SPME data from furfural analysis. We used the diffusion coefficient of furfural (*D* = 0.0872 cm^2^/s). *x* = fiber–sample distance; *t* = time of extraction; *C(x,t)* = concentration as a function of the fiber–sample distance for the extraction time.

The solution of such an equation is well known in the literature and may be expressed in terms of the single dimensionless variable *h* = *x*/√(4*Dt*) as
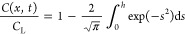
2In practice, since one can measure the amount of analytes absorbed during HS-SPME as a function of the fiber–sample distance we shall compare the theoretical prediction with the experimental observations. For this purpose, in Figure 5 we show the theoretical behavior of the concentration as a function of the *x* coordinate, i.e., the fiber–sample distance, using different extraction times. We found out that the concentration is more uniform for the longest extraction time (60 min), while for the shortest time (1 min) we observed a substantial coordinate dependence of the concentration over the relevant fiber–sample distance. In Figure 6 we compare the theoretical concentration obtained by solving Fick’s equation with the experimental data collected by the HS-SPME of furfural at an observation time of 10 min. Our theoretical data are rescaled to match the experimental data expressed in mAU at the lowest fiber–sample distance. From this comparison, we conclude that the variation of the analyte concentration predicted by the simple theoretical model discussed above agrees qualitatively with the experimental measurements. This reveals the importance of the fiber–sample distance as a relevant physical parameter in HS-SPME analysis.

## Conclusions

The dynamics of VOCs in HS is complex and therefore requires one to keep all of the parameters identical, mostly when HS-SPME is performed in pre-equilibrium conditions. The results obtained during this study, supported by a theoretical approach based on the diffusion equation, allowed us to highlight the importance of the fiber–sample distance as a crucial parameter to be considered during HS-SPME analyses. The impact of fiber–sample distance is specific to each VOC according to its distribution ratio (*K*) and diffusion constant (*D*). This parameter, never assessed before, should thus be considered in HS-SPME applications in order to limit operator-related variations by reporting more reproducible methods.

## Supporting Information Available

Chemicals and reagents, HS-SPME experimental conditions, gas chromatography-mass spectrometry (GC-MS) conditions, statistical analyses; HS-SPME conditions of 200 research articles published in 2019; experimental conditions used in GC-MS analyses; analyses of “primo fiore” wine samples; analyses of “Tavernello” wine samples; analyses of caciocavallo cheese; analyses of tea samples; analyses of chicken; analyses of a mixture of 6 volatile organic compounds; analyses of a mixture of 6 VOCs (PDF)
