# Supplementary material for: Fiber–Sample Distance, An Important Parameter To Be Considered in Headspace Solid-Phase Microextraction Applications
Source: Anal Chem. 2020 May 8;92(11):7478–84. doi: 10.1021/acs.analchem.9b05386 (PMC8007069; doi:10.1021/acs.analchem.9b05386)
Supplement: Supplementary file 1 — ac9b05386_si_001.pdf [file ac9b05386_si_001.pdf]

Supporting Information

Fiber-sample distance, an important parameter to be considered in headspace solid-phase microextraction (HS-SPME) applications.

Franks Kamgang Nzekoue[a], Simone Angeloni[a], Giovanni Caprioli[a], Manuela Cortese[b], Filippo Maggi[a], Umberto Marini Bettolo Marconi[c], Andrea Perali[a], Massimo Ricciutelli[b], Gianni Sagratini[a], Sauro Vittori[a]*.

[a]School of Pharmacy, and [b]HPLC-MS Lab, University of Camerino, via Sant'Agostino 1; [c]School of Sciences and Technology, University of Camerino, Via Madonna delle Carceri, 62032 Camerino, Italy.

* **Corresponding Author**: sauro.vittori@unicam.it.

Table of contents:

- Materials and methods:
  - Chemicals and reagents
  - HS-SPME experimental conditions
  - Gas chromatography-mass spectrometry (GC-MS) conditions
  - Statistical analyses.
- Tables and Figure:
  - Table S1
  - Table S2
  - Table S3
  - Table S4
  - Table S5
  - Table S6
  - Table S7
  - Figure S1
  - Figure S2

MATERIALS AND METHODS

Chemicals and reagents

Analytical standards of 3-methylbutanal (CAS. 590-86-3, MW 86.13 g/mol), 5-hexen-1-ol (CAS. 821-41-0, MW 100.16 g/mol), furfural (CAS. 98-01-1, MW 96.08 g/mol), linalool (CAS. 78-70-6, MW 154.25 g/mol), guaiacol (CAS. 90-05-01, MW 124.14 g/mol), pentane (CAS. 109-66-0, MW 72.15 g/mol), octadecane (CAS. 593-45-3, MW 254.49 g/mol), butanoic acid (CAS. 107-92-6, MW 88.11 g/mol), hexanoic acid (CAS. 142-62-1, MW 116.16 g/mol), decanoic acid (CAS. 334-48-5, MW 172.26 g/mol), 3-methylbutanoic acid (CAS. 503-74-2, MW 102.13 g/mol), D-limonene (CAS. 5989-27-5, MW 136.23 g/mol) and monobasic sodium phosphate (CAS. 10,049-25-5, MW 137.99 g/mol) were purchased from Sigma-Aldrich (Milan, Italy). Octanoic acid (CAS. 124-07-2, MW 144.21 g/mol) was supplied by Fluka (Germany).

Standard stock solutions were prepared in Milli-Q water (Millipore, Bedford, MA, USA) and stored at 4˚C. Individual working solutions were obtained by suitable dilution of standard stock solutions with Milli-Q water to obtain the desired concentrations.

HS-SPME experimental conditions

All extractions were carried-out using an autosampler PAL RSI 85 (Zwingen, Switzerland) allowing to reach the specific fiber penetration depths and reduce operator bias, working in accurate conditions with higher repeatability. Samples incubation were performed in a heat agitator set at an agitation speed of 250 rpm, with an on-time of 5 s and an off-time of 2 s. All the SPME fibers used were supplied by Supelco, (Bellefonte, PA, USA) and have a needle length of 1 cm, which was kept constant in all experiments. Fibers were inserted in HS vials at a speed of 20 mms^{-1} and were conditioned before and after extraction in a desorption port at 260˚C for 10 min.

Moreover, HS-SPME were also performed at higher stirring rates (400 and 550 rpm) to assess the effect of fiber-sample distance in higher agitation conditions. This range of stirring rates cover 79.6% of the HS-SPME experiments published in 2019. Indeed, in 200 research articles published in 2019, 51.5% did not specify the stirring rate, 17% were performed at 250 rpm, 11.5% were performed at 500 rpm, while 7% were performed without agitation, 3% were performed at 350 rpm, 3% at 600 rpm, 1% at 400 rpm, and 5.5% of the studies were performed using a stirring rate higher than 700 rpm (**Table S1**).

Stirring rates higher than 550 rpm were not advisable to avoid the fiber getting wet and damaged by the sample.

20 mL vial was chosen for HS-SPME because it is the most used vial volume in HS-SPME analysis. Once again, in 200 articles of 2019 in which HS-SPME was performed, more than 60% used a 20 mL vial (**Table S1**).

Fiber-sample distances

The fiber-sample distance refers to the distance between the tip of the SPME fiber and the sample surface. For a practical reason it is easier for the reader and the scientific community to indicate

either the depth of penetration of the fiber, which can be given with precision by an autosampler, or to precise the distance between the fiber tip and the top/surface of the sample (fiber-sample distance), which can be given by measuring this distance when extraction is performed manually.

Gas chromatography-mass spectrometry (GC-MS) conditions

GC-MS analyses were carried-out on an Agilent 7890B gas chromatograph system equipped with an autosampler (PAL RSI 85) and coupled to an Agilent 5977B mass selective detector (Santa Clara, California, USA). The carrier gas used was Helium, at a flow rate of 1.2 mL min^{-1}. After extraction, the fiber was desorbed in an injection port equipped with a liner (800 µL) at a temperature of 260°C under splitless mode. The inlet penetration depth was 40 mm, at a speed of 100 mm s^{-1}. The MSD transfer line was set at a temperature of 260°C. The MS source and quadrupole temperatures were 230 and 150°C, respectively. The gain factor was set at 0.05, while the resulting electron multiplier (EM) voltages were lower than 1300 V.

All VOCs separation was performed using a DB – Wax column (60 m, 0.25 mm i.d., 0.25 µm film thickness) (J&W Scientific, Folsom, CA, USA) following different oven temperature program gradients.

For the analyses of alkanes, the oven was maintained at 50°C for 4 min, then raised to 325°C at a rate of 15°C min^{-1}, and held at 325°C for 4 min. Data were acquired in scan ion mode with a scan range of 29-400 *m/z*.

For the analyses of the 6 VOCs, the oven temperature was kept at 35°C for 4 min, then raised to 120°C at a rate of 5°C min^{-1}, to 250°C at a rate of 16°C $min^{-1}$and held at 250°C for 1 min. For FFAs standards analyses, the initial oven temperature was 50°C for 3 min and increased at a rate of 5°C min^{-1} up to 150°C, held 1 min and then increased until 250°C at a rate of 10°C min^{-1} with a hold time of 7 min. MS operated in electron impact (E.I) mode and data were acquired in selected ion monitoring (SIM) mode. The selected SIM ions and time conditions for each compound are reported in **Table S2**.

For food samples analyses, oven temperature was maintained at 35°C for 4 min, then raised with a rate of 2.5°C min^{-1} to 120°C and increased at a rate of 15 °C min^{-1} to 250°C, held for 4 min. Data were acquired in scan, operating in E.I mode with a scan range of 29-400 *m/z*.

Compounds identification was based on the comparison of their GC retention times and mass spectra with analytical standards and with reference mass spectra from the US National Institute of Standards and Technology (NIST, 2017). Data were analyzed by using MSD ChemStation software (Agilent, Version G1701DA D.01.00).

Statistical analysis

For food samples analyses, results were expressed in percentage and were obtained by semi-quantification dividing the peak areas of the analyte of interest by the sum of the peak areas of all the identified compounds. On the other side, in standard samples analyses, results were expressed either as response factors (R.F = analyte peak area / reference compound peak area) or analyte peak areas. The results obtained from each analysis were validated by determining the S.D and the

relative standard deviation (RSD % = 100 x S.D / mean). Results with RSD values ≤ 10% were considered reliable. The student *t*-test was used to evaluate if the differences obtained between the tested penetration distances were statistically reliable. Probability values ≤ 0.05 ($p < 0.05$) were considered statistically significant.

Table S1. HS-SPME conditions of 200 research articles published in 2019. NS: non specified.

	Articles	Stirring speed (rpm)	Temp (°C)	Time (min)	Vial volume (mL)
1	Food Chemistry, Volume 281, 30 May 2019, Pages 49-56	0	37	40	NS
2	Food Research International, Volume 120, June 2019, Pages 285-294	0	50	60	10
3	Food Research International, Volume 123, September 2019, Pages 75-87	0	55	60	NS
4	LWT, Volume 112, September 2019, Article 107648	0	80	60	15
5	Analytica Chimica Acta, Volume 1081, 12 November 2019, Pages 72-80	0	25	26	4
6	Food Research International, Volume 123, September 2019, Pages 650-661	0	70	10	20
7	LWT Volume 11, 5November 2019, Article 108439	0	60	60	20
8	Food Chemistry Volume 285, 1 July 2019, Pages 147-155	0	NS	2	NS
9	Food Chemistry, Volume 275, 1 March 2019, Pages 143-153	0	50	45	15
10	Biological Control, Volume 133, June 2019, Pages 103-109	0	25	120	500
11	Food Packaging and Shelf Life, Volume 22, December 2019, Article 100412	0	60	40	NS
12	Food Chemistry, Volume 295, 15 October 2019, Pages 72-81	0	NS	15	4500
13	Food Chemistry Volume 283, 15 June 2019, Pages 579-587	0	25	45	20
14	Food Chemistry, Volume 282, 1 June 2019, Pages 153-163	0	35	20	40
15	Food Research International, Volume 120, June 2019, Pages 92-101	80	50	45	20
16	Environmental Pollution Volume 249June 2019Pages 305-310	200	55	30	75
17	Microchemical Journal Volume 145March 2019Pages 979-987	200	NS	30	20
18	Journal of Agricultural and Food Chemistry 67(49), pp. 13694-13705	200	60	30	20
19	Food Research International, Volume 121, July 2019, Pages 730-737	250	60	20	20
20	Food Research International, Volume 123, September 2019, Pages 684-696	250	60	30	20
21	Food Chemistry, Volume 270, 1 January 2019, Pages 518-526	250	45	20	5
22	Food Chemistry, Volume 280, 15 May 2019, Pages 83-95	250	60	50	20
23	Journal of Chromatography A, In press, corrected proof, Available online 22 October 2019, Article 460647	250	30	30	NS
24	Analytica Chimica Acta, In press, corrected proof, Available online 20 December 2019	250	55	30	20
25	LWT, Volume 115, November 2019, Article 108425	250	40	30	20
26	Food and Chemical Toxicology, Volume 134, December 2019, Article 110829	250	50	25	10
27	Journal of Chromatography A, In press, corrected proof, Available online 1 October 2019, Article 460584	250	45	50	20
28	Food Chemistry, Volume 287, 30 July 2019, Pages 313-323	250	25	10	15
29	Journal of Chromatography A, Volume 1601, 13 September 2019, Pages 60-70	250	55	30	20
30	Food Chemistry, Volume 290, 30 August 2019, Pages 16-23	250	37	5	NS

**31**	Microchemical Journal, Volume 145, March 2019, Pages 942-950	250	30	6	10
**32**	Waste Management, Volume 96, 1 August 2019, Pages 1-8	250	60	30	20
**33**	Helion, Volume 5, Issue 6, June 2019, Article e01842	250	60	30	10
**34**	Journal of Functional Foods, Volume 54, March 2019, Pages 271-280	250	45	120	NS
**35**	LWT, Volume 103, April 2019, Pages 186-191	250	30	40	20
**36**	Food Packaging and Shelf Life, Volume 21, September 2019, Article 100371	250	60	20	50
**37**	Food Chemistry Volume 27115 January 2019Pages 639-649	250	45	30	20
**38**	Food Research International Volume 115January 2019Pages 65-72	250	40	15	20
**39**	Food Chemistry Volume 2701 January 2019Pages 344-352	250	50	30	20
**40**	International Journal of Food Microbiology, Volume 311, 2 December 2019, Article 108350	250	50	45	NS
**41**	International Dairy Journal, Volume 96, September 2019, Pages 21-28	250	45	45	20
**42**	Food Packaging and Shelf Life, Volume 21, September 2019, Article 100328	250	50	30	20
**43**	LWT Volume 113, October 2019, Article 108258	250	60	50	NS
**44**	LWT Volume 111, August 2019, Pages 429-435	250	60	50	NS
**45**	Journal of Functional Foods, Volume 52, January 2019, Pages 81-89	250	60	50	NS
**46**	Food Chemistry, Volume 293, 30 September 2019, Pages 8-14	250	50	50	20
**47**	Food Chemistry, Volume 291, 1 September 2019, Pages 49-58	250	60	30	20
**48**	Science of The Total Environment, Volume 693, 25 November 2019, Article 133635	250	NS	NS	40
**49**	Foods 8(12),651	250	40	30	20
**50**	Journal of Chromatography A, Volume 1603, 11 October 2019, Pages 262-268	300	40	10	10
**51**	Food Chemistry, Volume 275, 1 March 2019, Pages 282-291	300	60	45	20
**52**	Food Research International, Volume 119, May 2019, Pages 369-377	300	40	30	NS
**53**	Journal of Chromatography A, Volume 1605, 8 November 2019, Article 360341	350	75	55	20
**54**	Molecules, 24(24), 4515	350	50	10	20
**55**	The International Journal of Biochemistry & Cell Biology, Volume 108, March 2019, Pages 40-50	350	50	30	NS
**56**	LWT, Volume 101, March 2019, Pages 113-122	400	50	60	20
**57**	Journal of Agricultural and Food Chemistry 67(47), pp. 13150-13163	400	45	20	20
**58**	Food Chemistry, Volume 292, 15 September 2019, Pages 75-80	500	43	45	20
**59**	Innovative Food Science & Emerging Technologies, Volume 53, May 2019, Pages 63-69	500	40	30	20
**60**	Food and Chemical Toxicology, Volume 130, August 2019, Pages 61-67	500	70	20	20
**61**	Talanta, Volume 191, 1 January 2019, Pages 535-544	500	30/60	60	20
**62**	Innovative Food Science & Emerging Technologies, Volume 56, August 2019, Article 102177	500	60	5	20
**63**	Food Research International, Volume 120, June 2019, Pages 514-522	500	30	40	20

**64**	Food and Chemical Toxicology, Volume 132, October 2019, Article 110647	500	40	15	20
**65**	Food Chemistry, Volume 285, 1 July 2019, Pages 39-45	500	80	15	NS
**66**	Microchemical Journal, Volume 150, November 2019, Article 104094	500	40	45	20
**67**	Food Research International, Volume 119, May 2019, Pages 733-740	500	30	45	20
**68**	Food Research International, Volume 125, November 2019, Article 108548	500	55	30	NS
**69**	Food Research International, Volume 123, September 2019, Pages 722-731	500	40	20	20
**70**	Food Chemistry Volume 27415 February 2019Pages 137-145	500	60	15	20
**71**	International Journal of Refrigeration Vol Volume 106, October 2019, Pages 24-32	500	60	20	15
**72**	Innovative Food Science & Emerging Technologies, Volume 58, December 2019, Article 102213	500	30	10	10
**73**	Food Research International, Volume 119, May 2019, Pages 554-563	500	45	40	20
**74**	Journal of Dairy Science, Volume 102, Issue 1, January 2019, Pages 202-210	500	50	10	20
**75**	Food Research International, Volume 122, August 2019, Pages 56-65	500	40	30	20
**76**	Journal of Food Engineering, Volume 260, November 2019, Pages 22-29	500	40	30	10
**77**	Innovative Food Science & Emerging Technologies, Volume 54, June 2019, Pages 64-77	500	40	5	10
**78**	LWT Volume 111, August 2019, Pages 1-8	500	60	50	15
**79**	Journal of Agricultural and Food Chemistry 67(49), pp. 13420-13429	500	45	45	NS
**80**	Separations, 6(4),46	500	45	40	20
**81**	Food Control, Volume 100, June 2019, Pages 335-349	550	40	30	NS
**82**	Journal of Chromatography A, Volume 1588, 15 March 2019, Pages 17-24	600	80	30	20
**83**	Atlanta, Volume 200, 1 August 2019, Pages 415-423	600	65	20-60	20
**84**	LWT, Volume 109, July 2019, Pages 83-92	600	40	30	20
**85**	Scientia Horticulture Volume 25227 June 2019Pages 121-129	600	40	40	20
**86**	Food Research International, Volume 125, November 2019, Article 108568	600	40	30	15
**87**	Journal of Chromatography A, Volume 1592, 10 May 2019, Pages 9-18	700	50	30	40
**88**	Food Chemistry Volume 301, 15 December 2019, Article 125252	700	50	60	15
**89**	Analytica Chimica Acta, In press, corrected proof, Available online 3 December 2019	750	NS	NS	20
**90**	Microchemical Journal, In press, corrected proof, Available online 29 November 2019, Article 104459	750	50	40	NS
**91**	Journal of Chromatography B, Volume 1133, 1 December 2019, Article 121824	750	NS	NS	10
**92**	Journal of Chromatography A, Volume 1591, 26 April 2019, Pages 55-61	750	100	45	20
**93**	Polymer Testing, Volume 73, February 2019, Pages 94-103	800	70	50	20
**94**	Food Chemistry, Volume 291, 1 September 2019, Pages 187-198	1000	60	45	20

**95**	Journal of Chromatography A, Volume 1603, 11 October 2019, Pages 92-101	1000	35	45	NS
**96**	Journal of Chromatography A, Volume 1602, 27 September 2019, Pages 142-149	1000	25	30	20
**97**	Arabian Journal of Chemistry, Volume 12, Issue 8, December 2019, Pages 1934-1944	1275	25	30	NS
**98**	Food Chemistry, Volume 301, 15 December 2019, Article 125264	NS	70	30	NS
**99**	Industrial Crops and Products, Volume 127, January 2019, Pages 225-231	NS	45	5	40
**100**	Food Chemistry, Volume 272, 30 January 2019, Pages 723-731	NS	60	40	20
**101**	Microchemical Journal, Volume 149, September 2019, Article 103991	NS	20	15	20
**102**	Microchemical Journal, Volume 148, July 2019, Pages 643-651	NS	50	30	15
**103**	LWT, Volume 104, May 2019, Pages 38-44	NS	40	55	20
**104**	Food Chemistry, Volume 277, 30 March 2019, Pages 753-765	NS	35	25	20
**105**	Meat Science, Volume 151, May 2019, Pages 43-53	NS	40	45	20
**106**	LWT, Volume 102, March 2019, Pages 304-309	NS	50	50	20
**107**	LWT, Volume 112, September 2019, Article 108256	NS	40	30	20
**108**	Food Chemistry, Volume 283, 15 June 2019, Pages 566-578	NS	30	75	90
**109**	Food Research International, Volume 121, July 2019, Pages 765-775	NS	40	70	20
**110**	Food Research International, Volume 119, May 2019, Pages 23-33	NS	60	80	15
**111**	Food Research International, Volume 125, November 2019, Article 108611	NS	60	50	15
**112**	LWT, Volume 112, September 2019, Article 108264	NS	45	30	15
**113**	LWT, Volume 108, July 2019, Pages 268-276	NS	40	40	20
**114**	LWT, Volume 99, January 2019, Pages 328-345	NS	80	30	20
**115**	Journal of Chromatography A, Volume 1599, 16 August 2019, Pages 17-24	NS	75	15	10
**116**	Food Research International, Volume 123, September 2019, Pages 550-558	NS	80	70	20
**117**	LWT, Volume 108, July 2019, Pages 221-232	NS	80	30	20
**118**	Food Chemistry, Volume 293, 30 September 2019, Pages 151-160	NS	45	30	20
**119**	Food Research International, Volume 116, February 2019, Pages 767-777	NS	38	60	100
**120**	Scientia Horticulturae, Volume 250, 10 May 2019, Pages 207-213	NS	25	40	20
**121**	Microchemical Journal, Volume 149, September 2019, Article 104064	NS	60	15	20
**122**	Analytical Biochemistry, Volume 578, 1 August 2019, Pages 36-44	NS	37	50	20
**123**	Journal of Functional Foods, Volume 59, August 2019, Pages 261-271	NS	40	35	10
**124**	Microchemical Journal, Volume 146, May 2019, Pages 1026-1032	NS	35	20	15
**125**	Food Research International, Volume 119, May 2019, Pages 84-98	NS	60	35	20
**126**	Food Research International, Volume 123, September 2019, Pages 481-502	NS	75	50	20
**127**	Flora, Volume 258, September 2019, Article 151428	NS	25	50	10
**128**	Atlanta, Volume 192, 15 January 2019, Pages 486-491	NS	60	30	10

**129**	Atlanta, Volume 202, 1 September 2019, Pages 90-95	NS	60	30	20
**130**	Microchemical Journal, Volume 146, May 2019, Pages 986-996	NS	60	65	100
**131**	Journal of Chromatography A, Volume 1594, 7 June 2019, Pages 45-53	NS	70	20	15
**132**	Food Research International, Volume 120, June 2019, Pages 620-630	NS	60	60	15
**133**	Journal of Plant Physiology, Volume 240, September 2019, Article 152994	NS	45	20	20
**134**	Microchemical Journal, In press, corrected proof, Available online 16 December 2019, Article 104532	NS	NS	NS	20
**135**	Science of The Total Environment, Volume 669, 15 June 2019, Pages 160-167	NS	70	60	10
**136**	Food Research International, Volume 119, May 2019, Pages 196-206	NS	60	50	10
**137**	Food Chemistry, Volume 278, 25 April 2019, Pages 406-414	NS	40	60	20
**138**	Journal of Chromatography A, Volume 1607, 6 December 2019, Article 460398	NS	100	25	22
**139**	Food Research International, Volume 125, November 2019, Article 108625	NS	40	60	20
**140**	Crop Protection, Volume 124, October 2019, Article 104839	NS	60	35	10
**141**	Food Chemistry, Volume 285, 1 July 2019, Pages 347-354	NS	30	60	N. S
**142**	Scientia Horticulturae, Volume 244, 26 January 2019, Pages 257-262	NS	60	105	20
**143**	Science of The Total Environment, Volume 681, 1 September 2019, Pages 392-399	NS	60	50	20
**144**	Atlanta, Volume 198, 1 June 2019, Pages 193-199	NS	45	15	10
**145**	Analytica Chimica Acta, Volume 1047, 24 January 2019, Pages 1-8	NS	NS	NS	20
**146**	International Journal of Food Microbiology, Volume 292, 2 March 2019, Pages 83-90	NS	60	60	20
**147**	Postharvest Biology and Technology, Volume 152, June 2019, Pages 127-138	NS	40	40	10
**148**	Talanta, Volume 205, 1 December 2019, Article 120080	NS	45	15	N. S
**149**	Food Research International Volume 122August 2019Pages 10-15	NS	50	30	20
**150**	Safety and Health at Work Volume 10, Issue 1March 2019Pages 114-121	NS	110	3	20
**151**	Journal of Bioscience and Bioengineering Volume 127, Issue 6June 2019Pages 710-713	NS	35	40	20
**152**	Food Research International Volume 120June 2019Pages 83-91	NS	45	45	20
**153**	Food Chemistry Volume 27415 February 2019Pages 118-122	NS	50	35	15
**154**	LWT Volume 113October 2019Article 108326	NS	37	NS	10
**155**	Food Chemistry Volume 27230 January 2019Pages 39-48	NS	60	50	20
**156**	International Journal of Food Microbiology Volume 3112 December 2019Article 108314	NS	40	20	20
**157**	Food Bioscience Volume 27February 2019Pages 30-36	NS	55	55	15
**158**	LWT Volume 108July 2019Pages 400-406	NS	50	70	20
**159**	Food Quality and Preference Volume 78December 2019Article 103735	NS	40	25	20
**160**	Food Chemistry Volume 28, 915 August 2019Pages 340-350	NS	60	70	15
**161**	Food Chemistry, Volume 279, 1 May 2019, Pages 356-363	NS	RT	60	15

**162**	Journal of Environmental Management, Volume 249, 1 November 2019, Article 109426	NS	70	15	15
**163**	Journal of Chromatography AIn press, corrected proof Available online 26 November 2019, Article 460739	NS	50	40	20
**164**	Food and Chemical Toxicology Volume 134December 2019Article 110833	NS	60	40	20
**165**	Food Research International, Volume 125, November 2019, Article 108531	NS	50	60	15
**166**	International Journal of Food Microbiology, Volume 290, 2 February 2019, Pages 86-95	NS	40	25	20
**167**	LWT Volume 108, July 2019, Pages 214-220	NS	45	45	15
**168**	Food Chemistry Volume 276, 15 March 2019Pages 572-582	NS	80	23	20
**169**	Postharvest Biology and Technology, Volume 154, August 2019, Pages 11-20	NS	40	60	20
**170**	Plant Science, Volume 287, October 2019, Article 110187	NS	50	30	20
**171**	Food Research International, Volume 119, May 2019, Pages 152-160	NS	50	60	20
**172**	Food Chemistry, Volume 287, 30 July 2019, Pages 186-196	NS	40	60	250
**173**	Journal of Cereal Science, Volume 85, January 2019, Pages 6-14	NS	60	60	20
**174**	Food Chemistry, Volume 289, 15 August 2019, Pages 215-222	NS	45	50	20
**175**	Food Chemistry, Volume 290, 30 August 2019, Pages 135-143	NS	60	55	15
**176**	Reproductive Toxicology, Volume 84, March 2019, Pages 114-121	NS	NS	NS	20
**177**	Food Research International, Volume 121, July 2019, Pages 593-603	NS	50	45	15
**178**	Journal of Chromatography A, Volume 1597, 19 July 2019, Pages 132-141	NS	50	30	20
**179**	Food Research International, Volume 122, August 2019, Pages 318-329	NS	45	40	20
**180**	Food Chemistry, Volume 289, 15 August 2019, Pages 645-656	NS	40	60	15
**181**	Heliyon Volume 5, Issue 6June 2019, Article e01953	NS	60	30	20
**182**	LWT, Volume 104, May 2019, Pages 8-15	NS	60	30	NS
**183**	LWT, Volume 111, August 2019, Pages 211-217	NS	40	60	NS
**184**	Journal of Food Engineering, Volume 259, October 2019, Pages 12-20	NS	40	30	NS
**185**	Food Chemistry, Volume 286, 15 July 2019, Pages 659-668	NS	45	20	20
**186**	International Journal of Food Microbiology, Volume 299, 16 June 2019, Pages 8-22	NS	37	50	40
**187**	Microchemical Journal, Volume 145, March 2019, Pages 1119-1128	NS	60	60	20
**188**	Food Chemistry, Volume 277, 30 March 2019 Pages 84-95	NS	50	60	120
**189**	The Journal of Supercritical Fluids, Volume 143, January 2019, Pages 211-222	NS	50	10	22
**190**	Food Chemistry, Volume 271, 15 January 2019, Pages 298-308	NS	60	30	10
**191**	LWT, Volume 101, March 2019, Pages 145-151	NS	50	60	100
**192**	Food Microbiology, Volume 77, February 2019, Pages 166-172	NS	40	30	20
**193**	Postharvest Biology and Technology, Volume 153, July 2019, Pages 43-51	NS	50	60	10
**194**	Food Chemistry, Volume 292, 15 September 2019, Pages 227-236	NS	60	30	15
**195**	Biological Control, Volume 129, February 2019, Pages 195-200	NS	NS	NS	15

**196**	Food Chemistry, Volume 274, 15 February 2019, Pages 915-924	NS	60	60	20
**197**	Food Chemistry, Volume 289, 15 August 2019, Pages 250-258	NS	35	60	20
**198**	LWT, Volume 105, Mai 2019, Pages 48-56	NS	55	55	40
**199**	Food Analytical Methods 12(11), pp. 2582-2590	NS	50	60	20
**200**	Food Research International, Volume 119, May 2019, Pages 477-491	NS	50	45	10

Table S2. Experimental conditions used in GC-MS analyses. Time windows and selected ions for each monitored compound in selected ion monitoring mode (SIM).

Groups	Compounds	Time windows (min)	SIM Ions (***m/z***)
Study on Free fatty acids			
1	Butanoic acid	13 – 24.5	73; 88
2	Isovaleric acid	24.5 – 27	60; 87
3	Hexanoic acid	27 – 29	73; 87
4	Octanoic acid	29 – 32	101; 115
5	Decanoic acid	32 – 40	129; 172
Study on mixtures of 6 VOCs standards*			
1	3-Methylbutanal	0 – 13	44; 58
2	Hexen-1-ol	13 – 23.3	67; 82
3	Furfural	23.3 – 24.5	96; 95
4	Furfuryl acetate	24.5– 24.8	98; 140
5	Linalool	24.8 – 28.1	71; 93
6	Guaiacol	28.1 –30.1	109; 124

* VOCs: Volatile organic compounds.

Table S3. Analyses of *"primo fiore"* wine samples: percentage (%) of each identified volatile organic compound (VOC) obtained at two fiber exposition depths (20 and 40 mm) at different temperatures (30, 50 and 80°C). Values are expressed as mean ± standard deviation. % = 100 x peak area analyte / total peak area. *: Differences were statistically significant for the VOCs considered ($p \leq 0.05$).

VOCs	30°C		50°C		80°C	
	**20 mm**	**40 mm**	**20 mm**	**40 mm**	**20 mm**	**40 mm**
Ethyl acetate	12.4 ± 0.4	8.4 ± 0.08*	8.2 ± 0.2	5.8 ± 0.07*	8.0 ± 0.7	5.8 ± 0.7
Ethanol	53.2 ± 4.1	37.9 ± 0.5*	53.4 ± 0.6	43.7 ± 0.1*	58.9 ± 0.2	52.1 ± 0.16*
Ethyl butanoate	1.0 ± 0.05	1.8 ± 0.1*	1.3 ± 0.03	1.3 ± 0.01	0.7 ± 0.1	0.6 ± 0.2
1-Propanol, 2 methyl	0.8 ± 0.01	0.8 ± 0.01	1.0 ± 0.02	0.8 ± 0.01*	0.8 ± 0.1	0.7 ± 0.02
Isoamyl acetate	5.1 ± 0.2	11.1 ± 0.01*	4.7 ± 0.5	4.0 ± 0.01	2.5 ± 0.03	1.9 ± 0.02*
1-butanol, 3-methyl	9.6 ± 0.3	11.0 ± 0.01*	10.8 ± 1.0	10.2 ± 0.01	8.6 ± 0.5	8.4 ± 0.4
Ethyl caproate	8.8 ± 0.6	10.0 ± 0.01	6.9 ± 0.3	9.6 ± 0.01*	4.7 ± 0.2	4.1 ± 0.2
Hexyl acetate	0.6 ± 0.03	1.4 ± 0.01*	0.3 ± 0.1	1.1 ± 0.00*	0.2 ± 0.02	0.2 ± 0.03
Ethyl lactate	0.5 ± 0.03	1.6 ± 0.02*	1.2 ± 0.02	1.5 ± 0.00*	1.7 ± 0.04	1.5 ± 0.03*
1-Hexanol	0.3 ± 0.02	1.1 ± 0.02*	0.5 ± 0.01	1.1 ± 0.00*	0.8 ± 0.02	0.6 ± 0.01*
Ethyl octanoate	7.4 ± 0.6	7.4 ± 0.00	5.7 ± 0.3	6.5 ± 0.00	2.4 ± 0.3	5.0 ± 0.2*
Acetic acid	-	0.3 ± 0.00*	0.2 ± 0.05	0.3 ± 0.01	0.6 ± 0.06	0.5 ± 0.03
Ethyl decanoate	1.3 ± 0.2	1.6 ± 0.01	1.0 ± 0.03	1.5 ± 0.00*	0.3 ± 0.02	0.7 ± 0.07*
Ethyl succinate	0.2 ± 0.02	0.6 ± 0.00*	0.5 ± 0.01	1.2 ± 0.00*	1.0 ± 0.07	1.6 ± 0.04*
Ethyl decenoate	0.06 ± 0.01	0.1 ± 0.01*	0.07 ± 0.00	0.1 ± 0.00*	0.2 ± 0.04	0.2 ± 0.03
Acetic acid, 2-phenylethyl ester	0.05 ± 0.00	0.1 ± 0.00*	0.1 ± 0.01	0.4 ± 0.01*	0.2 ± 0.01	0.7 ± 0.1*
Hexanoic acid	1.9 ± 0.1	1.3 ± 0.4	0.6 ± 0.01	2.8 ± 0.2*	1.4 ± 0.01	2.3 ± 0.4
Phenylethyl alcohol	0.6 ± 0.04	1.1 ± 0.03*	0.9 ± 0.01	1.5 ± 0.01*	1.4 ± 0.1	1.9 ± 0.03*
Octanoic acid	0.6 ± 0.02	1.9 ± 0.2*	1.9 ± 0.2	4.6 ± 0.02*	4.4 ± 0.3	7.5 ± 0.2*
Decanoic acid	0.06 ± 0.00	0.5 ± 0.1*	0.5 ± 0.07	2.0 ± 0.02*	1.2 ± 0.1	3.4 ± 0.07*
Sum of peak areas of identified VOCs (x 10^{6})	1452 ± 25	1929 ± 3*	1699 ± 64	1984 ± 2*	1726 ± 32	1855 ± 15*

Table S4. Analyses of "*Tavernello*" wine samples: percentage (%) of each identified volatile organic compound (VOC) obtained at two fiber exposition depths (20 and 40 mm) after 20 min at 35°C. Values are expressed as mean ± standard deviation. % = 100 x peak area analyte / total peak area. *: Differences were statistically significant for the VOCs considered ($p \leq 0.05$).

	20mm	40mm
	Mean± std dev (%)	Mean± std dev (%)
Ethanol	91.66 ± 0.05	91.29 ± 0.01*
3-Methyl-1-butanol	6.01 ± 0.04	6.40 ± 0.05*
Ethyl butyrate	0.10 ± 0.00	0.11 ± 0.00*
Isoamyl acetate	1.92 ± 0.00	1.86 ± 0.05
Gamma-butyrolactone	0.05 ± 0.00	0.08 ± 0.01*
Hexyl acetate	0.07 ± 0.00	0.07 ± 0.00
Ethyl octanoate	0.18 ± 0.00	0.18 ± 0.00
Ethyl decanoate	0.00 ± 0.00	0.01 ± 0.00*
Sum of peak areas of identified VOCs	$602 \pm 22 \times 10^{6}$	$777 \pm 15 \times 10^{6}$*

Table S5. Analyses of caciocavallo cheese. Percentage (%) of each identified volatile organic compound (VOC) obtained at two fiber exposition depths (20 and 40 mm) after 30 min of extraction at 45°C.

Values are expressed as mean ± standard deviation (std dev). % = 100 x peak area analyte / total peak area. *: Differences were statistically significant for the VOCs considered ($p \leq 0.05$).

	20mm	40mm
	Mean± std dev (%)	Mean± std dev (%)
2-Heptanone	0.50 ± 0.02	0.76 ± 0.06*
D-Limonene	0.52 ± 0.01	0.89 ± 0.02*
3-Methyl-1-butanol	0.15 ± 0.02	0.19 ± 0.03
Acetoin	2.16 ± 0.17	5.60 ± 0.28*
2-propanone, 1-hydroxy	0.05 ± 0.00	0.13 ± 0.00*
Acetic acid	0.08 ± 0.00	4.69 ± 0.55*
Benzaldehyde	0.17 ± 0.00	0.23 ± 0.00*
Butanoic acid	59.93 ± 2.15	46.72 ± 0.11*

Isovaleric acid	0.54 ± 0.00	1.27 ± 0.06*
Pentanoic acid	1.06 ± 0.07	2.76 ± 0.15*
Hexanoic acid	32.91 ± 2.12	33.32 ± 0.54
Octanoic acid	1.93 ± 0.13	3.44 ± 0.27*
Sum of peak areas of identified VOCs	245 ±14 x 10 ^{6}	476±17 x 10^{6}*

Table S6. Analyses of tea samples. Percentage (%) of each identified volatile organic compound (VOC) obtained at two fiber exposition depths (20 and 40 mm) after 40 min of extraction at 50°C. Values are expressed as mean ± standard deviation (std dev). % = 100 x peak area analyte / total peak area. *: Differences were statistically significant for the VOCs considered ($p \leq 0.05$).

	**20 mm**	**40 mm**
	Mean ± std dev (%)	Mean ± std dev (%)
2-Methylbutanal	4.5 ± 0.3	4.0 ± 0.3
Pentanal	3.1 ± 0.3	3.2 ± 0.3
2-Ethylfuran	2.6 ± 0.4	2.9 ± 0.3
2-Pentenal	1.8 ± 0.3	1.5 ± 0.2
Hexanal	24.2 ± 2.3	35.5 ± 1.6*
Furfural	2.6 ± 0.4	2.5 ± 0.3
2-Hexenal	29.8 ± 3.2	26.6 ± 0.8
Heptanal	2.6 ± 0.4	2.2 ± 0.3
2-Heptenal	6.5 ± 1.2	4.2 ± 0.8
Benzaldehyde	11.4 ± 1.8	8.4 ± 0.6
6-Methylhept-5-en-2-one	4.3 ± 0.8	3.0 ± 0.5
2,4-Heptadienal	6.6 ± 0.7	5.7 ± 0.3
Sum of peak areas of identified VOCs	19.5 ± 0.4 x 10^{6}	22.7 ± 0.9 x 10^{6}*

Table S7. Analyses of chicken. Percentage (%) of each identified volatile organic compound (VOC) obtained at two fiber exposition depths (20 and 40 mm) after 30 min of extraction at 40°C.

Values are expressed as mean ± standard deviation (std dev). % = 100 x peak area analyte / total peak area. *: Differences were statistically significant for the VOCs considered ($p \leq 0.05$).

	**20 mm**	**40 mm**
	Mean± std dev (%)	Mean± std dev (%)
2-Methyl-1-propanol	2.3 ± 0.6	1.52 ± 0.14
Nonane	0.5 ± 0.01	1.35 ± 0.10*
Heptanone	0.6 ± 0.03	0.94 ± 0.20
3-methyl-1-butanol	25.3 ± 5.4	36.50 ± 1.19
Acetoin	33.5 ± 4.2	18.40 ± 1.95*
Nonanone	0.8 ± 0. 2	1.15 ± 0.06
Leucicacid	6.15 ± 0.2	15.18 ± 1.85*
2-ethyl-1-Hexanol	1.15 ± 0.02	1.01 ± 0.07
Phenylehylalcohol	8.22 ± 1.77	8.30 ± 0.51
Phenol	21.53 ± 0.07	13.31 ± 0.30*
Sum of peak areas of identified VOCs	1328 ± 5 x 10^5	2550 ± 2 x 10^5*

Figure S1.

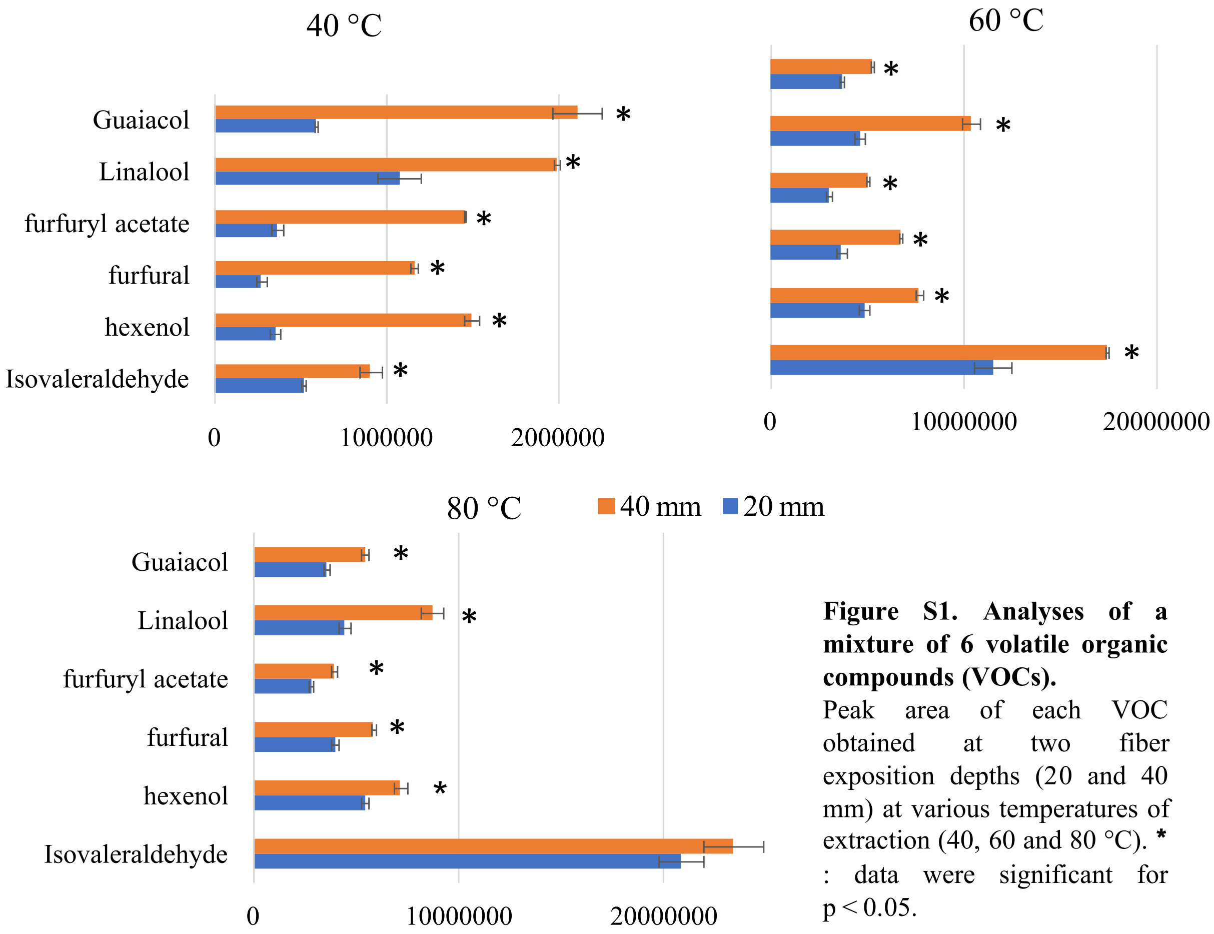

Figure S1. Analyses of a mixture of 6 volatile organic compounds (VOCs).
Peak area of each VOC obtained at two fiber exposition depths (20 and 40 mm) at various temperatures of extraction (40, 60 and 80 °C). * : data were significant for $p < 0.05$.

Figure S2.

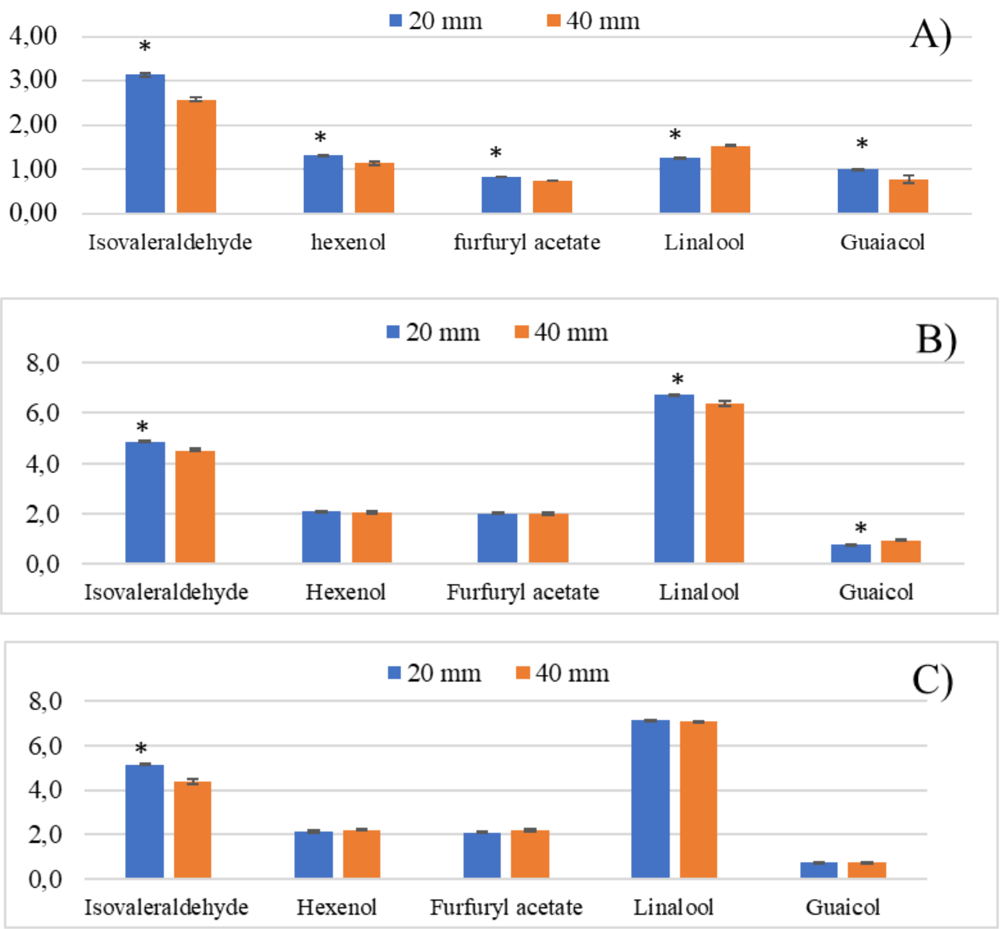

Figure S2. Analyses of a mixture of 6 volatile organic compounds (VOCs). Response factors (RF) obtained at two fiber penetration distances (20 and 40 mm) at various stirring rate of extraction (fixed time of extraction: 15 min and temperature: 60 °C).

A) Stirring rate: 250 rpm;

B) Stirring rate: 400 rpm;

C) Stirring rate: 550 rpm.

RF= analyte peak area/furfural peak area. *****: data were significant for $p < 0.05$.

In a study performed on a mixture of VOCs, the RF differences was statistically significant for all 5 compounds at 250 rpm. With the increase of the stirring speed, the variations were reduced, as expected, but remained statistically significant for some analytes (**Figure S2**).

It is important to note that the aims of this study were neither to determine the best stirring rate for HS-SPME experiments, nor the right sample-fiber distance. Our aim was simply to demonstrate that in pre-equilibrium conditions, as for several published papers, it is important to specify the sample-fiber distance or the fiber penetration depth in order to increase the reproducibility of the researches reported in literature.

In addition, few researchers have started very recently to take into consideration this parameter, giving info in their papers about the distance between the fiber tip and the sample surface or the fiber penetration depths (Food Research International, Volume 123, September 2019, Pages 650-661; Food Packaging and Shelf Life, Volume 22, December 2019, Article 100412; Food Chemistry, Volume 292, 15 September 2019, Pages 227-236).
